# Physicochemical and mechanical comparison of denture base materials after artificial aging

**DOI:** 10.1186/s12903-026-08745-1

**Published:** 2026-06-02

**Authors:** Dania Hany M. Elazzouni, Muhammad Sohail Zafar, Abdulaziz Abdullah Alkhureif, Leonel S. J. Bautista, Abdulsalam Abdullah Alnasser, Aftab Ahmed Khan

**Affiliations:** 1 Oral & Maxillofacial Prosthodontics Department, Faculty of Dentistry, King Abdulaziz University, Jeddah, Saudi Arabia; 2 Department of Clinical Sciences, College of Dentistry, Ajman University, Ajman, United Arab Emirates; 3 Centre of Medical and Bio-allied Health Sciences Research, Ajman University, Ajman, United Arab Emirates; 4 School of Dentistry, University of Jordan, Amman, Jordan; 5 Dental Health Department, College of Applied Medical Sciences, King Saud University, Riyadh, Saudi Arabia; 6 Dental and Oral Rehabilitation Research Department, College of Dentistry, King Saud University, Riyadh, Saudi Arabia

**Keywords:** Denture base, 3D-printing, CAD/CAM milling, Artificial aging, Physicochemical properties

## Abstract

**Background:**

This study aimed to evaluate the impact of artificial aging on the surface integrity, mechanical properties, and thermal stability of three commercially available denture base materials: conventional heat-polymerized polymethyl methacrylate (PMMA), CAD/CAM-milled PMMA, and 3D-printed photopolymerizable resin.

**Methods:**

Cylindrical samples (6 mm diameter × 3 mm height) were fabricated for each material. All samples were finished, and measurements were performed on the finished flat surface. A total of 20 samples were prepared for each material; half were tested at baseline and half after artificial aging, yielding a final sample size of *n* = 10 per group. Artificial aging was performed using 5000 thermocycles between 5 °C and 55 °C with a 10-second dwell time. Surface roughness (mean Ra, µm) was measured using non-contact optical profilometry, while surface microhardness (mean Vickers hardness, HV) was assessed using the Vickers hardness test. Thermal stability was evaluated using thermogravimetric analysis (TGA; mass loss, %) and differential scanning calorimetry (DSC; heat flow, mW/mg). Surface morphology was examined through scanning electron microscopy (SEM).

**Results:**

Results indicated a significant increase in surface roughness for all materials post-aging (*p* < 0.05), with 3D-printed resin showing the greatest change (0.04 → 0.07 μm) and milled PMMA the least (0.04→0.06 μm). Milled PMMA had the highest baseline hardness (20.01 HV) and exhibited the least reduction after aging, while 3D-printed resin experienced the most pronounced decline (16.96→16.21 HV). TGA showed mass gain in conventional PMMA (+ 0.23%) and 3D-printed resin (+ 0.13%), whereas milled PMMA lost mass (-0.16%).

**Conclusions:**

Overall, CAD/CAM-milled PMMA demonstrated superior thermal aging resistance, maintaining hardness and surface quality, followed by conventional PMMA. In contrast, 3D-printed resin was most vulnerable to surface deterioration and softening under artificial aging. CAD/CAM-milled PMMA can be a recommended choice for the denture base.

## Background

The long-term clinical success of removable prostheses largely depends on the physicochemical stability, mechanical durability, and surface integrity of denture base materials [1]. Polymethyl methacrylate (PMMA) remains the most widely used material for conventional denture bases due to its acceptable aesthetics, ease of processing, and cost-effectiveness [2]. However, the inherent limitations of conventional PMMA, such as poor impact resistance, dimensional instability, and susceptibility to surface degradation over time [3, 4], have driven the development of alternative fabrication techniques and materials in recent years [5].

Computer-aided design and computer-aided manufacturing (CAD/CAM) technologies have revolutionized prosthodontic workflows by introducing subtractive (milled) and additive (3D-printed) methods for fabricating denture bases [6]. Milled denture bases, typically manufactured from pre-polymerized PMMA blocks, offer enhanced polymerization homogeneity, reduced residual monomer content, and improved mechanical properties [5, 7]. In contrast, 3D printing techniques, particularly those utilizing photopolymerizable resins, allow for rapid, customizable, and cost-effective fabrication [8]. Yet, concerns remain about the long-term durability of printed resins, particularly their resistance to surface degradation and thermal stress.

Significant differences in mechanical properties, dimensional stability, and color stability among conventional, CAD/CAM-milled, and 3D-printed denture base materials have been reported in previous studies. Helal et al. demonstrated significantly higher dimensional stability for CAD/CAM-milled denture bases compared with conventional and 3D-printed counterparts [9]. Similarly, Yu et al. reported superior mechanical properties, including flexural strength and modulus, for CAD/CAM-milled materials compared with conventional and 3D-printed materials [10]. In contrast, Alp et al. observed comparable color stability and surface roughness between CAD/CAM-milled and conventional denture base materials after 5000 coffee thermocycles [11]. Despite these findings, considerable variability exists across the literature regarding fabrication techniques and evaluation protocols. Moreover, the combined assessment of surface, mechanical, and thermal properties following standardized artificial aging remains limited.

Thermal fluctuations in the oral environment, particularly through the intake of hot and cold food and beverages, can induce cyclic thermal stresses on denture base materials [12], potentially compromising their structural and surface properties over time [13–15]. Thermocycling is a widely accepted method for simulating these intraoral conditions, providing valuable insight into the durability of dental polymers [16]. Despite the rapid adoption of digital fabrication techniques, comparative evidence on the artificial aging behavior of conventional, 3D-printed, and milled denture base materials remains limited. To date, only a small number of investigations have compared conventional, milled, and 3D-printed denture base materials, and these often differ in methodology, making direct comparisons under consistent conditions difficult.

Understanding the impact of artificial aging on denture base materials is essential for predicting their long-term clinical performance and guiding prosthodontic material selection. The present study aimed to evaluate the effects of artificial aging on three commercially available denture base materials: conventional heat-polymerized PMMA, CAD/CAM-milled PMMA, and 3D-printed resin. The null hypothesis was that artificial aging would not produce significant differences in the surface, mechanical, or thermal properties among these materials.

## Methods

### Sample preparation

Interacryl Hot (Interdent, Opekarniska, Slovenia) was used to fabricate samples for the heat-polymerized acrylic group. The powder-to-liquid ratio of 2.3:1.0 (by weight) was mixed manually in a rubber-mixing bowl using a stainless-steel spatula until the mixture reached a dough-like consistency. The material was then packed into a cylindrical gypsum mold within a dental flask. A load of 100 N was applied to eliminate excess material and ensure proper compaction. Polymerization was performed in a water bath maintained at 73 °C for 8 h, followed by a terminal curing phase at 100 °C for 1 h.

Cylindrical samples were prepared from pre-polymerized denture base resin blocks (Vipi Block Gum, VIPI, São Paulo, Brazil), designed for CAD/CAM applications. The blocks were sectioned using an automated cutting unit (Multi Slicer ASM420M, Okamoto, Nagoya, Japan) fitted with a diamond blade (SD400, Noritake, Nagoya, Japan). The cut segments were then shaped into cylindrical samples to obtain uniform geometry suitable for mechanical testing. No additional CAD/CAM milling surface treatment was performed, as the objective was to evaluate the intrinsic properties of the pre-polymerized resin block material under standardized surface finishing conditions applied equally to all groups.

A liquid resin with a medium pink color (Atos, SmartDent, São Paulo, Brazil) was used in a stereolithographic printer (Asiga Max UV, Asiga, Australia). Disc-shaped samples were designed in dental CAD software (Exocad, Exocad GmbH, Darmstadt, Germany) before printing. Samples were arranged using a standard nesting strategy to ensure uniform printing conditions and minimize variability between builds. Discs were printed at a 90° build angle with a layer thickness of 50 μm. Supports were automatically generated by the printer software where required and were carefully removed using rotary instruments after printing. After printing, samples were immersed in isopropyl alcohol for 5 min, air-dried, and post-cured in a UV chamber (LC-3DPrint Box, NextDent) for 30 min. Post-curing was performed using LED light sources with a wavelength range of approximately 315–550 nm under controlled temperature conditions according to the manufacturer’s specifications for 30 min.

**Fig. 1:**
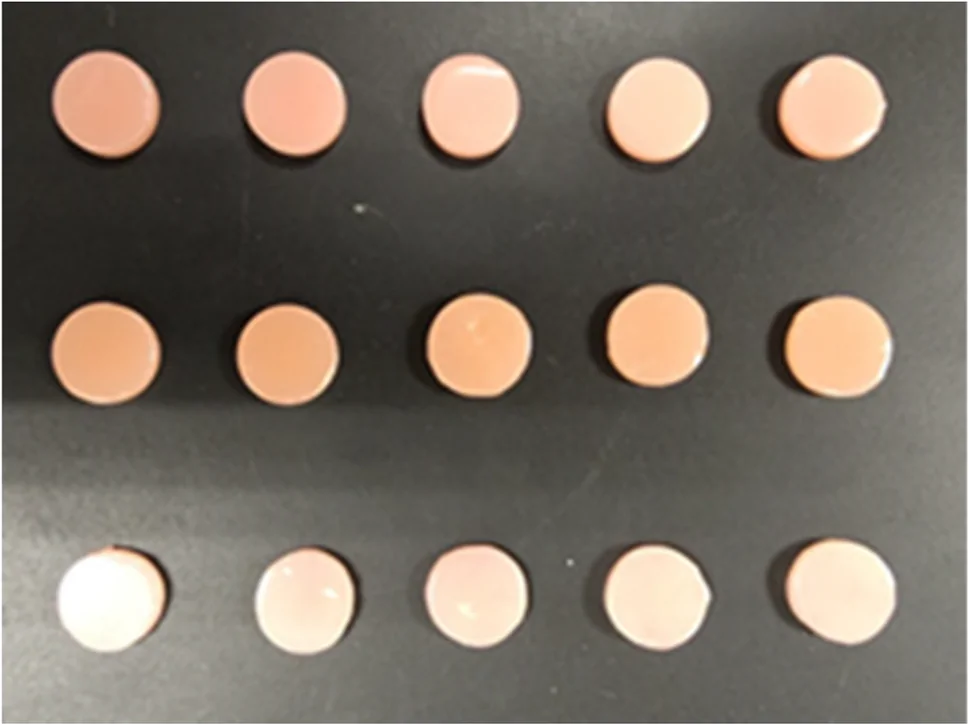
Representative image of cylindrical samples used in the study. The top row shows conventional heat-polymerized PMMA discs, the middle row displays CAD/CAM-milled PMMA discs, and the bottom row presents 3D-printed photopolymerizable resin discs

For all three denture base materials, samples were standardized to cylindrical dimensions of 6 mm in diameter and 3 mm in height (Fig. 1). A total of 80 samples were fabricated for each material group, with 10 samples per group allocated for each experimental test under both baseline and aged conditions. All samples were finished using 2000-grit silicon carbide paper (3 M, USA) under continuous water irrigation to produce smooth, standardized surfaces on the flat circular area designated for testing. All samples were finished by a single operator following the same protocol to minimize variability. Next, the specimens of each group were stored in a desiccator for 24 h before testing to ensure complete drying and stabilization.

Next, all samples were immersed in distilled water at 37 °C for 48 h to simulate intraoral hydration. For each material and for each experimental test, ten samples were allocated to the baseline group, while the remaining ten samples were assigned to the aged group. The aged samples were subjected to 5000 thermocycles between 5 °C and 55 °C, with a dwell time of 10 s in each bath, to simulate approximately six months of clinical aging [17]. To minimize bias, samples were randomly allocated using a computer-generated randomization sequence, and investigators were blinded to groups during testing or analysis.

### Surface roughness (Ra) measurement

Surface roughness of the samples was quantitatively assessed using a three-dimensional optical profilometry system (ContourGT, Bruker, Germany), employing a non-contact methodology. The arithmetical mean roughness value (Ra) was acquired via white light interferometry using a 5× magnification objective in conjunction with a standard imaging unit. Each measurement was executed within a delineated region of 0.975 mm × 0.720 mm, precisely positioned at the center of the sample surface. Each sample was scanned at three separate locations on the finished surface, and the average of the three readings was recorded for each sample. Data acquisition and analysis were conducted under controlled conditions using VISION64 software (version 5.30, Bruker, Germany) [18].

### Microhardness testing

Vickers microhardness testing was performed using a microhardness tester (MMT–X7 Matsuzuwa, Japan) under a load of 300 g and a dwell time of 15 s. Five indentations were placed on each sample’s finished surface with a minimum spacing of 0.5 mm between indentations. The tester automatically calculated the Vickers Hardness Number (VHN) based on the standard formula [19]:

$$VHN=1.854\,F/d^{2}$$

where 𝐹 is the applied load (kgf) and 𝑑 is the average length of the two diagonals of the indentation (mm).

### Dynamic thermal analysis

Thermal characterization was conducted using simultaneous dynamic thermal analysis, combining thermogravimetric analysis (TGA) and differential scanning calorimetry (DSC) on an SDT Q600 system (TA Instruments, New Castle, DE, USA). Representative samples, each weighing between 4 and 8 mg, were placed within the instrument’s heating chamber. Thermal scanning was performed under a nitrogen atmosphere (flow rate: 20 mL/min) at a controlled heating rate of 10 °C per minute, ranging from ambient temperature (28 °C) to 600 °C. Mass changes were recorded as a function of temperature during TGA analysis, while heat flow signals were simultaneously acquired during the same thermal cycle for DSC analysis. The initial sample mass was normalized to 100%, and the residual mass (%) was calculated relative to it. Slight apparent increases above 100% may occur due to normalization procedures, buoyancy effects, or minor gas adsorption during thermal analysis. Following thermal exposure, specimens were allowed to cool to room temperature over 2 h. DSC analysis was performed to evaluate the thermal behavior of the materials through heat flow patterns during controlled heating; however, due to the amorphous nature of PMMA-based materials and limitations of the output, precise glass transition temperature (Tg) was not quantitatively determined. While thermal stability and mass variation were evaluated from the TGA curves using the integrated Q600 analysis software [20]. Residual mass (%) was calculated as the percentage of mass remaining at 600 °C relative to the initial mass at 28 °C. Mass change (%) was determined as the difference between the initial and final masses and expressed as a percentage of the initial mass.

### Scanning electron microscope (SEM) evaluation

Surface morphology was examined using SEM (JSM-6360LV, JEOL, Tokyo, Japan), operated in secondary electron detection mode at an accelerating voltage of 10 kV. Microstructural imaging of samples was performed at magnifications of 500× and 1000×. Image acquisition and analysis were facilitated through the use of proprietary software integrated with the SEM apparatus.

### Statistical analysis

The sample size was calculated using G*Power version 3.1 (Heinrich Heine University, Düsseldorf, Germany). An effect size of f = 0.40 was assumed for differences in the tested properties among materials and treatment conditions, with a significance level of α = 0.05 and a statistical power (1 − β) of 0.80. For a two-way ANOVA design (3 materials × 2 treatment conditions), the minimum required sample size was 8 samples per group. To account for potential sample loss, 10 samples were included in each group.

All quantitative data were analyzed using IBM SPSS Statistics software (v26.0, IBM Corp., USA). Normality of data was verified using the Shapiro–Wilk test. Intergroup comparisons were performed using two-way ANOVA, followed by Tukey’s post hoc test where applicable. A *p*-value < 0.05 was considered statistically significant.

## Results

**Fig. 2:**
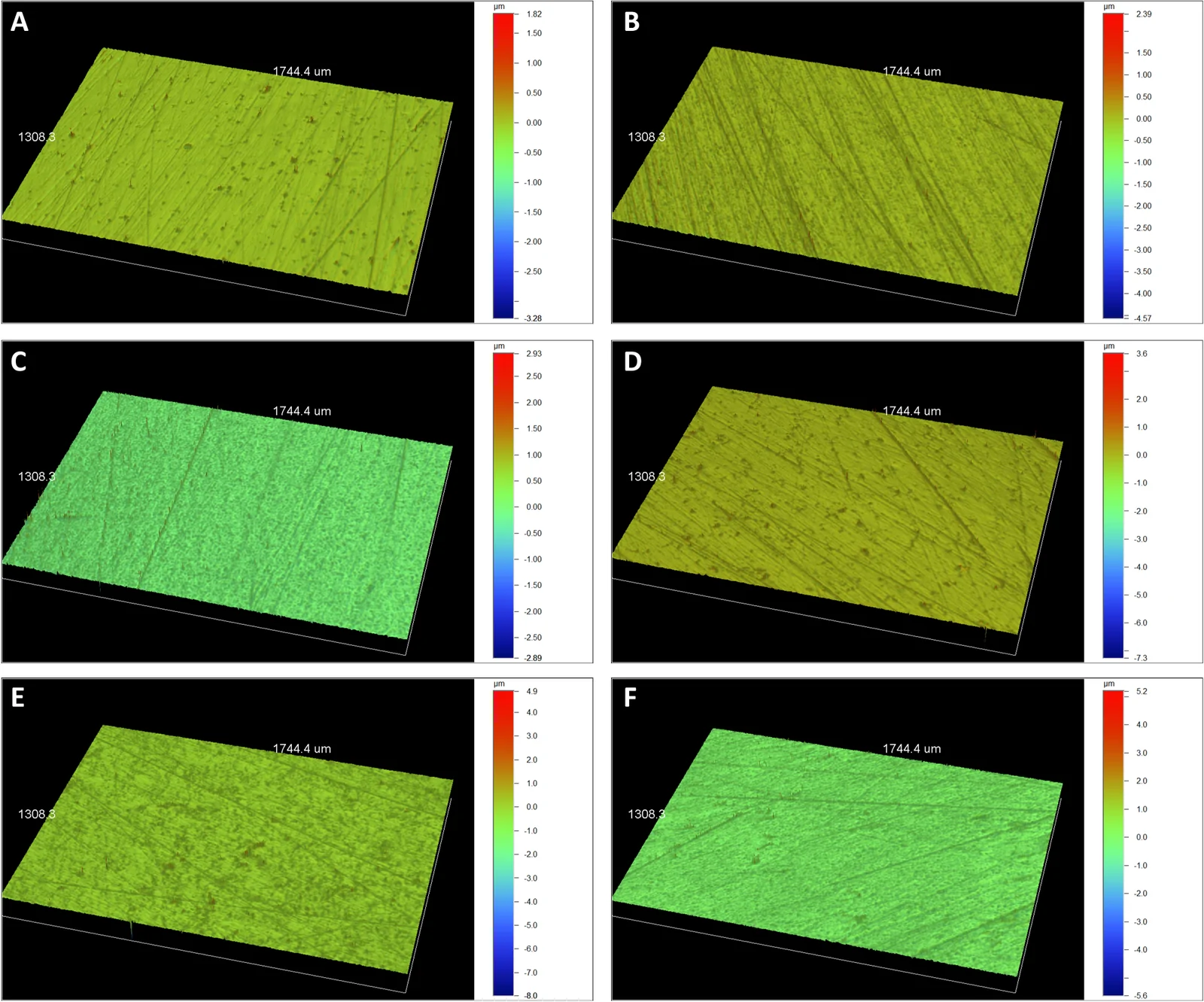
3D surface profilometry images showing surface topography of baseline (**A**–**C**) and aged (**D**–**F**) denture base materials. Images (**A**) and (**D**) represent the conventional denture base, (**B** and **E**) the milled denture base, and (**C** and **F**) the 3D printed denture base. Color gradients indicate surface height variations, with red representing peaks and blue representing valleys

Table 1 presents the surface roughness data (Ra, µm) of baseline and aged samples of conventional, milled, and 3D printed denture base materials. All materials showed an increase in surface roughness following the aging process. The conventional denture base exhibited the lowest surface roughness in its baseline state (0.03 μm), while the milled and 3D-printed materials showed slightly higher values (0.04 μm). After aging, the 3D printed material displayed the highest roughness (0.07 μm), indicating greater surface deterioration. Statistically significant differences were observed between the treatment conditions (baseline vs. aged) within each material (*p* < 0.05). The corresponding 3D surface roughness images visually support the quantitative data in Fig. 2. Images A–C represent the baseline conventional, milled, and 3D printed denture base samples, respectively, showing relatively smoother surfaces with fewer irregularities. In contrast, images D–F show the aged counterparts, where increased surface roughness and texture variation are apparent.

**Table 1:** Mean surface roughness (R_a_, µm) of baseline and aged samples of conventional, milled, and 3D printed denture base materials

Material	Surface roughness (*R*_a_, µm)	
	Baseline (Mean ± SD)	Aged (Mean ± SD)
Conventional Denture base	0.03 ± 0.01^A^_a_	0.05 ± 0.01^B^_a_
Milled Denture base	0.04 ± 0.01^A^_b_	0.06 ± 0.00^B^_b_
3D-Printed denture base	0.04 ± 0.00^A^_a, b_	0.07 ± 0.01^B^_b_

Key: Different superscript uppercase letters indicate significant differences between treatment conditions within the same material, while different subscript lowercase letters indicate significant differences between materials within the same treatment condition

Table 2 presents the Vickers hardness (HV) of baseline and aged conventional, milled, and 3D printed denture base materials. Among the baseline samples, the milled denture base exhibited the highest hardness (20.01 HV), followed closely by the conventional material (19.90 HV), while the 3D printed material showed the lowest hardness (16.96 HV). Aging led to a reduction in hardness in all materials, with the most notable decrease observed in the 3D-printed group (16.21 HV). Statistically significant differences were observed between materials within the same treatment condition (*p* < 0.05).

**Table 2:** Mean Vickers hardness (HV) of baseline and aged samples of conventional, milled, and 3D printed denture base materials

Material	Vickers microhardness (HV)	
	Baseline (Mean ± SD)	Aged (Mean ± SD)
Conventional Denture base	19.90 ± 0.70_a_	18.88 ± 0.43_a_
Milled Denture base	20.01 ± 0.37_a_	19.17 ± 0.93_a_
3D-Printed denture base	16.96 ± 0.22_b_	16.21 ± 0.49_b_

Key: Different lowercase subscript letters indicate statistically significant differences between materials within the same treatment condition. No statistically significant differences were observed between baseline and aged conditions within the same material (*p* > 0.05)

Table 3 presents the thermogravimetric behavior of baseline and aged denture base materials in terms of residual mass and mass change. Among baseline groups, all materials showed minimal mass loss, with the highest residual mass observed in the 3D printed group (100.25%), indicating a slight mass gain (+ 0.25%). After aging, the conventional and 3D printed materials exhibited a further increase in residual mass (+ 0.23% and + 0.13%, respectively), while the milled group showed a significant decrease (99.84%, -0.16%). Statistically significant differences were observed between materials within the same treatment condition (*p* < 0.05).

**Table 3:** Thermogravimetric analysis (TGA) data showing residual mass (%) and mass change (%) of baseline and aged conventional, milled, and 3D printed denture base materials

Material	Baseline (Mean ± SD)		Aged (Mean ± SD)	
	Residual mass (%)	Mass change (%)	Residual mass (%)	Mass change (%)
Conventional Denture base	99.94 ± 0.02_a_	-0.06 ± 0.02	100.23 ± 0.04_a_	+ 0.23 ± 0.04
Milled Denture base	99.97 ± 0.02_a_	-0.03 ± 0.02	99.84 ± 0.02_b_	**-**0.16 ± 0.02
3D-Printed denture base	100.25 ± 0.03_b_	+ 0.25 ± 0.03	100.13 ± 0.04_c_	+ 0.13 ± 0.04

Key: Different lowercase subscript letters indicate statistically significant differences between materials within the same treatment condition. No statistically significant differences were observed between baseline and aged conditions within the same material (*p* > 0.05)

**Fig. 3:**
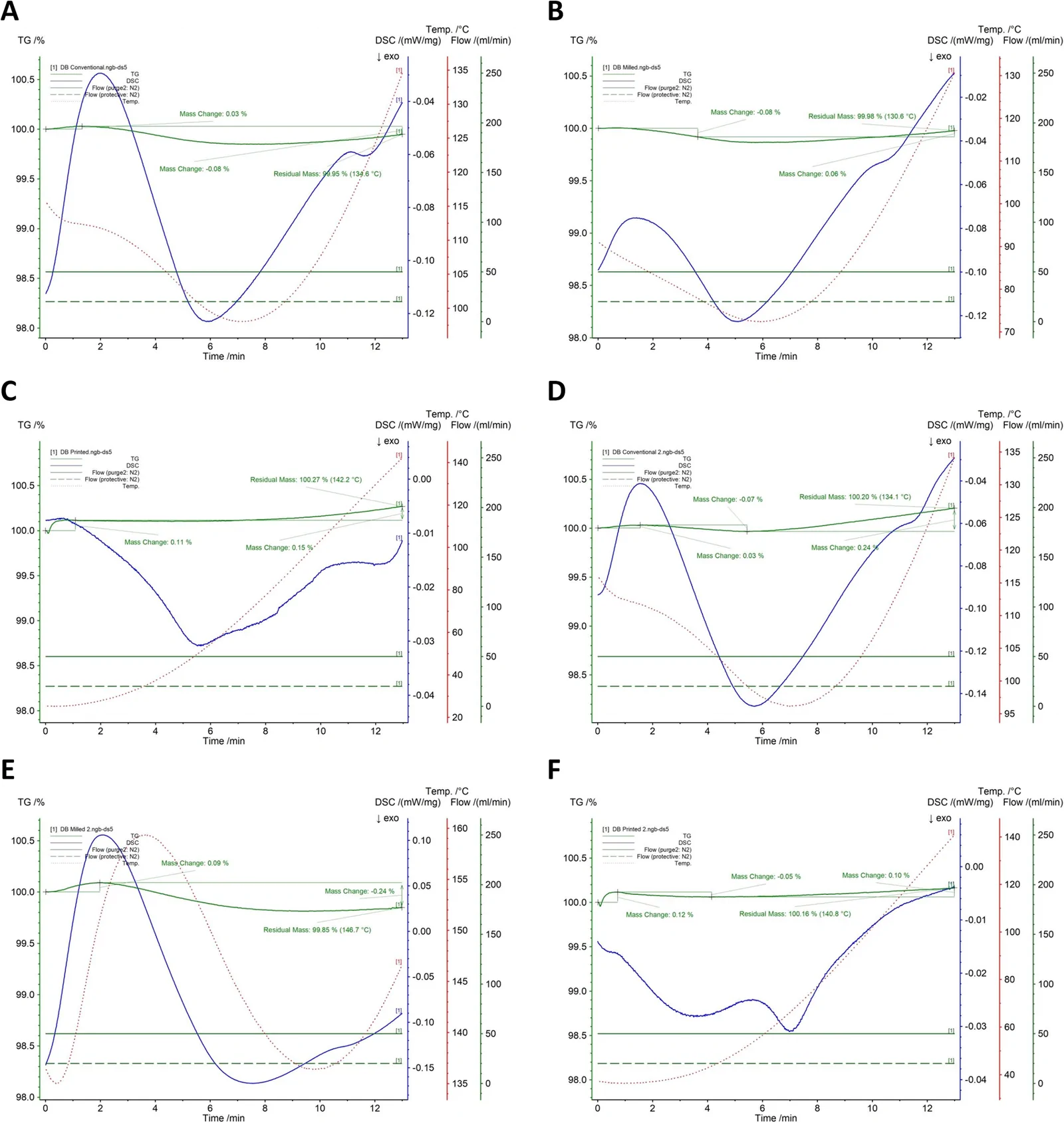
Thermogravimetric (TGA) and differential scanning calorimetry (DSC) curves of baseline (**A**–**C**) and aged (**D**–**F**) denture base materials

Figure 3 shows the thermogravimetric (TG) and differential scanning calorimetry (DSC) curves of conventional (A, D), milled (B, E), and 3D-printed (C, F) denture base materials in baseline (A–C) and aged (D–F) conditions. The TG curves (green) represent mass change, while the DSC curves (blue) show thermal transitions during heating under a nitrogen atmosphere. In the baseline state (A–C), minimal mass changes were observed across all materials, with the 3D printed denture base (C) exhibiting a slight positive mass change (+ 0.25%), possibly due to mild gas adsorption. The milled and conventional materials showed minor mass losses (-0.03% and − 0.06%, respectively). In the aged condition (D–F), a distinct divergence in thermal behavior is evident. The milled denture base (E) showed the greatest mass loss (-0.16%), suggesting thermal instability or degradation following aging. In contrast, both conventional (D) and 3D printed (F) materials displayed positive mass changes, consistent with the possibility of atmospheric interaction or surface reactivity during thermal exposure.

**Fig. 4:**
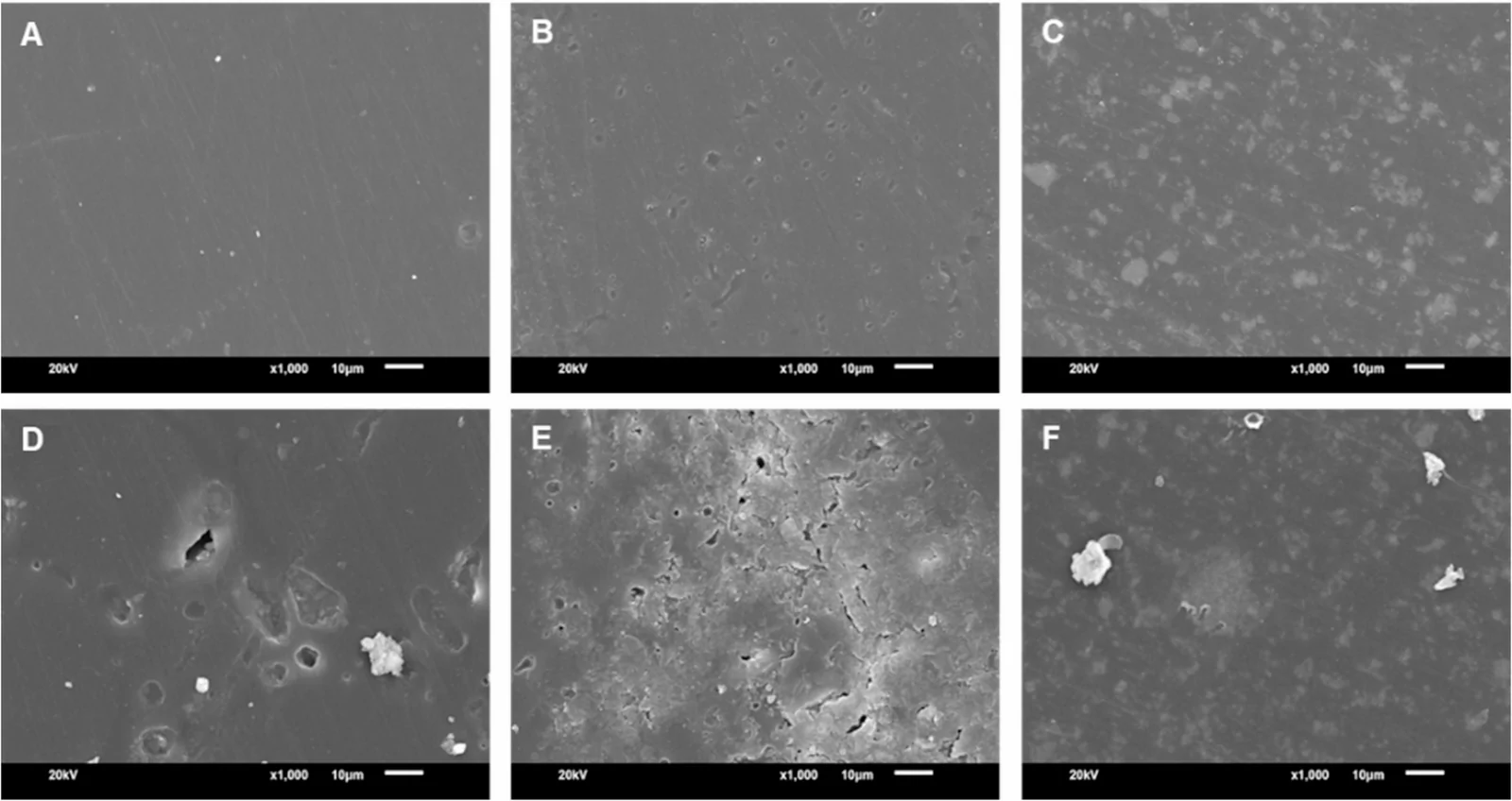
SEM images of denture base materials at 1,000× magnification: (**A**, **D**) conventional, (**B**, **E**) milled, and (**C**, **F**) 3D printed, shown in baseline condition (**A**–**C**) and after artificial aging (**D**–**F**)

Figure 4 presents SEM pictograms of conventional, milled, and 3D-printed denture base materials before (A–C) and after artificial aging (D–F). The baseline conventional denture base appeared relatively smooth with only minor imperfections (Fig. 4A), while the milled surface was also smooth with minor imperfections but exhibited fine lines or subtle marks, likely resulting from the milling process (Fig. 4B). However, the 3D-printed denture base material presented a rougher, more irregular texture (Fig. 4C). Following artificial aging, all materials exhibited increased surface roughness and the formation of pits, cracks, and debris, but the effect was most pronounced in the 3D-printed samples (Fig. 4F), which displayed severe degradation and irregularity.

## Discussion

This study comprehensively analyzed the aging effects on key physicochemical, mechanical, and surface properties of commercially available denture base materials: conventional heat-polymerized PMMA, CAD/CAM-milled PMMA, and 3D-printed resin. The findings elucidate the impact of contemporary manufacturing technologies on the long-term performance and durability of denture base materials, while also delineating the specific susceptibilities and advantages inherent to each fabrication approach. Overall, artificial aging significantly affected the evaluated properties of the denture base materials; therefore, the null hypothesis was rejected.

Surface roughness is a critical parameter in material evaluation, as increased roughness promotes the accumulation of biofilm and plaque, contributing to the development of caries and periodontal disease [21]. As expected, the aged group had higher surface roughness than the baseline group. This is because the thermal stress induces microcracks and surface erosion in polymeric denture bases [13]. Following artificial aging, all materials exhibited a statistically significant increase in surface roughness (*p* < 0.05), consistent with prior studies showing that thermal cycling and water absorption contribute to microstructural degradation and surface topographic changes in denture base resins [11, 22]. The most pronounced increase was observed in the 3D printed group (from 0.04 to 0.07 μm), suggesting greater susceptibility to hydrothermal stress, possibly due to lower cross-link density or residual monomer content in light-cured photopolymer systems [8]. The layer-by-layer curing in 3D printing creates a microscopically uneven texture due to incomplete polymer cross-linking and residual monomer, which is compounded by the stair-step effect inherent to the printing process. Furthermore, the post-processing protocols for 3D printed samples may be insufficient to fully eliminate surface irregularities, resulting in higher initial and aged roughness values than conventionally fabricated bases. Increased surface roughness promotes biofilm adhesion and aesthetic deterioration [5, 23], which may compromise long-term surface stability under repeated thermal stress.

Surface hardness is a key determinant in material selection [24], as denture bases undergo abrasive and mechanical stresses during clinical use and hygiene procedures [2]. Microhardness results revealed significantly higher baseline values for conventional and milled samples compared to the 3D-printed group. This aligns with previous studies demonstrating superior mechanical performance of pre-polymerized CAD/CAM blocks, attributed to high-pressure, high-temperature industrial polymerization and greater cross-link density, which enhance material homogeneity and hardness [23, 25]. While the conventional method employs heat polymerization at elevated temperatures (ranging from 74 °C to 100 °C), it facilitates the formation of a denser and more cohesive polymer network with reduced residual monomer content and fewer internal defects such as porosity or voids [26]. When processed using a long curing cycle or water bath method, conventional PMMA undergoes more complete polymerization, which positively influences its mechanical properties, including surface hardness [26]. Unlike 3D-printed resins, conventional PMMA does not possess interlayer interfaces, resulting in a more homogeneous structure. The observed reduction in microhardness following aging across all groups reflects water‑induced plasticization and progressive degradation of the denture base polymer network [27]. Water sorption during aging likely increased intermolecular spacing within the polymer matrix, facilitating chain mobility and reducing resistance to indentation. This effect would be more pronounced in materials with lower cross-link density and higher residual monomer content, such as photopolymerized 3D-printed resins [5].

TGA provided quantitative data on mass changes and thermal stability, while DSC offered qualitative insight into the thermal behavior of the materials. Both conventional and 3D-printed groups exhibited slight positive mass changes following aging, likely attributable to water sorption or atmospheric gas adsorption, phenomena previously reported in PMMA-based materials exposed to humid or oxidative environments [28]. In contrast, the milled PMMA group demonstrated a marginal loss, indicative of minor thermal degradation or volatilization processes potentially exacerbated by artificial aging. However, the overall superior thermal stability of milled PMMA aligns with existing literature, which attributes enhanced resistance to its industrial polymerization, resulting in high cross-link density and minimal residual monomer [23, 25]. Additionally, Tg values obtained reinforce the observations regarding polymer network stability. The DSC analysis indicated differences in thermal transition behavior among the materials, with 3D-printed samples exhibiting patterns suggestive of lower thermal stability, likely due to reduced cross-linking and higher residual monomer content, both of which attenuate thermal and mechanical resistance [29]. This thermal instability further highlights the need for optimized resin formulations in 3D printing technologies to match the durability of established PMMA materials.

The morphological data corroborate well with the surface roughness, microhardness, and physicochemical data. The superior initial smoothness of conventional PMMA may relate to meticulous laboratory finishing protocols, whereas milled surfaces retain subtle tooling marks (Figs. 2B and 3B). The pronounced surface pitting, cracking, and granular debris in aged 3D-printed resin (Fig. 4F) directly align with its significant increase in surface roughness and the hardness reduction, reflecting structural incohesion from incomplete polymerization and interlayer bonding strength. Milled PMMA’s moderate roughness elevation (0.04→0.06 μm) and hardness retention (20.01→19.17 HV) correspond to localized micro-scratches without catastrophic propagation (Fig. 3E), confirming its superior structural integrity. Physicochemically, TGA mass loss in milled PMMA (-0.16%) correlates with SEM-visible micro-fissures suggesting volatile release, while mass gain in conventional (+ 0.23%) and 3D-printed (+ 0.13%) materials aligns with hygroscopic swelling-induced microporosity.

In summary, the differing aging behaviors of the materials stem from fundamental variations in their polymerization and network structure. Industrially cured PMMA blocks used in subtractive manufacturing achieve high conversion rates and homogeneity, while the layer-by-layer photopolymerization of additive manufacturing introduces variability in adhesion and conversion. These inherent structural characteristics ultimately dictate the observed differences in surface degradation, hardness retention, and thermal stability.

The results confirm the superior in vitro durability of conventional and milled denture base materials, reflecting greater resistance to surface degradation, mechanical wear, and thermal stress. In contrast, 3D-printed resins, while advantageous for customization and rapid production, exhibited increased surface roughness and reduced hardness after aging, raising concerns about biofilm accumulation and long-term performance.

While informative, the in vitro aging model used in this study does not fully replicate the complex physiological and biochemical conditions of the oral cavity. Additionally, using a single artificial aging protocol may not fully reflect the multifactorial degradation processes found intraorally. The artificial aging protocol simulates temperature and humidity effects but does not account for mechanical forces, salivary enzymes, dietary chemical exposure, or microbial activity. As such, caution is warranted when extrapolating these findings to clinical performance. Future studies should incorporate cyclic mechanical loading to simulate masticatory stresses, biofilm challenge models to evaluate microbial adhesion and material degradation, and combined thermo-mechanical aging protocols to better replicate clinical service conditions. Long-term in vivo studies and multi-factorial in vitro simulations integrating cyclic loading, pH fluctuation, and microbial exposure are recommended to predict clinical longevity more accurately.

## Conclusions

Artificial aging significantly affected the properties of the denture base materials, leading to the rejection of the null hypothesis. The findings suggest that degradation patterns depend on the fabrication methodology. Overall, CAD/CAM-milled PMMA demonstrated the best performance, with superior mechanical durability and thermal stability compared to conventional and 3D-printed materials. Conventional PMMA provided a reliable all-around performance. In contrast, 3D-printed resins, despite their workflow advantages, exhibited pronounced susceptibility to surface and mechanical degradation under thermal stress. From a clinical perspective, CAD/CAM-milled PMMA is the recommended choice for the denture base. The CAD/CAM-milled PMMA offers enhanced longevity and thermal resistance; otherwise, conventional PMMA remains a reliable alternative.

## Data Availability

Raw data were generated at King Saud University, Riyadh; KSA. Derived data supporting the findings of this study are available from the corresponding author on request.

## Abbreviations

SEM: Scanning Electron Microscopy
DSC: Differential Scanning Calorimetry
TGA: Thermogravimetric Analysis
PMMA: Polymethyl Methacrylate
CAD: Computer-Aided Design
CAM: Computer-Aided Manufacturing
Ra: Surface Roughness
HV: Vickers Hardness
VHN: Vickers Hardness Number
Tg: Glass Transition Temperature
Tm: Melting Temperature
